# Trade-offs in the production of animal vocal sequences: insights from the structure of wild chimpanzee pant hoots

**DOI:** 10.1186/s12983-017-0235-8

**Published:** 2017-11-06

**Authors:** Pawel Fedurek, Klaus Zuberbühler, Stuart Semple

**Affiliations:** 1 Department of Primatology, Max Planck Institute for Evolutionary Anthropology, Leipzig, Germany; 2 Institute of Biology, University of Neuchâtel, Neuchâtel, Switzerland; 3 School of Psychology and Neuroscience, University of St Andrews, St Andrews, Scotland, UK; 4 Centre for Research in Evolutionary, Social and Interdisciplinary Anthropology, University of Roehampton, London, UK

**Keywords:** Acoustic trade-offs, Call sequences, Chimpanzee, Compression, Menzerath’s law, Pant hoot

## Abstract

**Background:**

Vocal sequences - utterances consisting of calls produced in close succession - are common phenomena in animal communication. While many studies have explored the adaptive benefits of producing such sequences, very little is known about how the costs and constraints involved in their production affect their form. Here, we investigated this issue in the chimpanzee (*Pan troglodytes schweinfurthii*) pant hoot, a long and structurally complex vocal sequence comprising four acoustically distinct phases – introduction, build-up, climax and let-down.

**Results:**

We found that in each of these phases, and for the sequence as a whole, there was a negative relationship between the number of calls produced and their average duration. There was also a negative relationship between the total duration of some adjacent phases. Significant relationships between the fundamental frequency of calls and their number or duration were found for some phases of the sequence, but the direction of these relationships differed between particular phases.

**Conclusions:**

These results indicate that there are trade-offs in terms of signal duration at two levels in pant-hoot production: between call number and duration, and between the relative durations of successive phases. These trade-offs are likely to reflect biomechanical constraints on vocal sequence production. Phase-specific trade-offs also appear to occur between fundamental frequency and call number or duration, potentially reflecting that different phases of the sequence are associated with distinct types of information, linked in different ways to call pitch. Overall, this study highlights the important role of costs and constraints in shaping the temporal and acoustic structure of animal vocal sequences.

**Electronic supplementary material:**

The online version of this article (10.1186/s12983-017-0235-8) contains supplementary material, which is available to authorized users.

## Background

Vocal signals are an integral part of animal communication and have important functions, ranging from attracting mating partners to coordinating activities between group members [1, 2]. Vocal sequences, utterances consisting of a series of calls produced in close succession, are common phenomena and found across a wide range of animal taxa [3]. The adaptive benefits of such signals have been widely researched. For example, repeated production of the same call type has been found to reduce the probability of signal misinterpretation by the receiver [4], while production of vocal sequences composed of different call types can enhance the communicative potential of individual calls or different combinations of calls [5–7], facilitate individual recognition [8], or play a role in attracting mates [9, 10] or repelling sexual rivals [11, 12].

While a range of adaptive benefits of vocal sequences have been demonstrated, much less attention has been paid to the potential costs and constraints involved in producing such signals. Although vocalising in itself has a metabolic cost, this appears to be relatively low [13–15]; however, the production of long vocal sequences may involve further energetic costs linked to the fine muscle control that is needed - over several levels of vocal production - to generate these complex utterances. Specifically, vocal sequence production may be affected by biomechanical constraints related to lung capacity, breathing control [16], airflow control at the source, and movements of the vocal tract [17, 18]. Additionally, a potential constraint on vocal sequence utterance is related to the risk of hyperventilation, which may occur if vocalisations are produced in too rapid succession [19]. These costs and constraints could lead to significant trade-offs in how vocal sequences are constructed.

A recent study of male gelada (*Theropithecus gelada*) vocal sequences provided evidence for just such a trade-off: a negative correlation was found between the number of calls in a sequence and the average duration of these constituent calls [20]. The production of sequences with a greater number of calls thus only appears possible if shorter calls are used within them, which may reflect energetic or breathing constraints on vocal production [20]. This pattern is consistent with Menzerath’s law, a linguistic law which states that the larger the construct, the smaller is the size of its constituents [20–23]. This law has been linked mathematically to compression - the information-theoretic principle of minimising code length - and it has been argued that this is a universal principle not only of animal behaviour [24], but also of biological information systems in the broadest sense [20].

In vocal sequences with distinct phases - such as orlotan bunting (*Emberiza hortulana*) song [25], rock hyrax (*Procavia capensis*) calls [11] or chimpanzee pant hoots [26] - another potential trade-off is in the overall investment of effort between phases. There is evidence that different phases in such sequences can be associated with different types of information, and be relevant to different potential receivers [11, 12, 27, 28]. Consequently, social factors such as audience composition may affect how signallers potentially benefit from allocating more effort to one phase or another. If energetic or breathing-related constraints apply to the whole sequence, individuals may benefit by allotting more to one phase at the cost of what is possible to allot to another, depending on their specific circumstances. While it has been shown that callers can modify the duration of specific phases or notes within a sequence [11, 29], it is unclear whether such adjustments at the level of whole phases affect the duration of other phases.

Duration - of calls or sequence phases - is, however, only one measure of cost, and constraints may apply to other, not necessarily temporal, acoustic features of vocal sequences. One spectral acoustic feature of calls that has been associated with energetic costs is fundamental frequency (F0) [30]. In a number of animals, including Japanese quails (*Coturnix japonica*) [31], Alston’s singing mice (*Scotinomys teguina*) [32] and humans (*Homo sapiens*) [33], low frequency of calls reflects good health or condition of the caller, partially because such calls are energetically costly to produce. However, in other animals, such as red deer (*Cervus elaphus*) [34], chacma baboons (*Papio cynocephalus ursinus*) [35] and white-handed gibbons (*Hylobates lar*) [36] producing high rather than low-frequency calls is associated with good quality among males. This could be because high-frequency calling requires a high sub-glottal pressure and elevated muscular effort, and therefore incurs metabolic costs, but more likely is because calling at high frequencies requires significant motor control of the larynx [17, 18, 37].

It is possible, therefore, that there is a trade-off between F0 on the one hand, and call duration or number on the other hand, with the nature of this trade-off depending on whether high or low frequency calls are more energetically costly. For example, if it is particularly costly to produce low frequency calls, it would be expected that the longer or more numerous are the calls in a sequence, the higher would be their F0. If producing calls of high frequency is especially costly, the opposite relationship should be expected. To our knowledge there have been no studies examining directly the possibility that there is a trade-off in vocal sequences between call pitch and either call duration or call number.

**Fig. 1:**
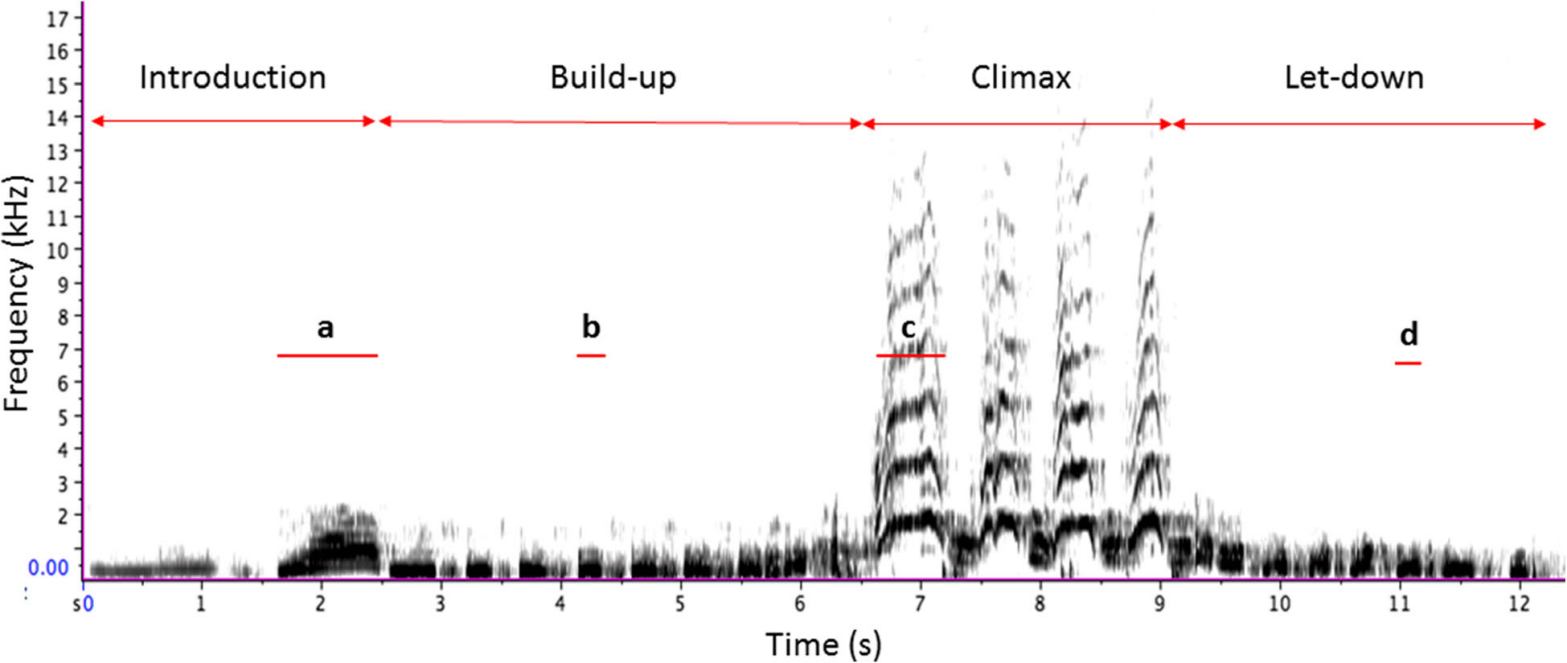
Spectrographic representation of a pant hoot, with the four phases and their calls. **a** – an introduction call, **b** – a build-up call, **c** – a climax call, **d** – a let-down call. In this example, the introduction consists of two calls, the build-up of nine calls, the climax of four calls, and the let-down of eight calls. Red lines below “**a**”, “**b**”, “**c**” and “**d**” represent durations of calls within the four phases

In this study, we tested for evidence of trade-offs in chimpanzee pant hoots. This complex vocal sequence consists of four distinct phases ([38]; Fig.1; see Additional file 1 for an example of a recording). Pant-hooting usually starts with the introduction phase, consisting of low-frequency and low-amplitude calls, which then grade into the build-up phase, consisting of a series of short, low-frequency calls [26]. The build-up, in turn, grades into the climax phase, the loudest part of the sequence that can include one or several ‘screams’ (i.e. climax calls). This is often followed by the let-down phase, which has similar acoustic features to the build-up phase [26]. There is considerable within- [29] and between- [38] individual variation in terms of the number of calls within all four phases of the sequence. Pant hoots have multiple social functions, ranging from signalling social status and bonds, to coordinating grouping and proximity [39–42], and recent evidence indicates that different phases fulfil different communicative functions [43].

To explore potential trade-offs in construction of this complex vocal sequence, we tested first for a negative relationship between call number and duration in each phase, and for the overall pant hoot. Next, we tested whether the durations of adjacent phases in the sequence are negatively related. Finally, we tested whether in each phase, F0 is related to call number or duration; for this analysis there was no clear expectation as to the direction of relationship, as it is unclear whether low- or high-frequency calling is particularly costly for male chimpanzees [44, 45].

## Methods

### Study site and study subjects

The study was carried out on the Sonso chimpanzee community of Budongo Forest, Uganda. The group has been continuously observed since 1990 and is well habituated to the presence of human observers [46]. At the time of the study, the community contained 75 individuals, with a core home range of around 15 km^2^. Study subjects were adult (*N* = 11: ≥ 16 years) and late adolescent (*N* = 2: ≥ 13–15 years; [47]) males. See Additional files 2, 3, 4 and 5 for information on study males’ age, their dominance rank, and the number of pant hoot recordings per individual.

### Sampling methods

Fieldwork was conducted between June and October 2013, February and September 2014 and January and December 2015. Data were collected between 0700 and 1630 h local time. Data collection methods for this study were entirely non-invasive.

Each day, an arbitrarily chosen male was followed for the whole day. Pant hoots were audio-recorded from the focal male and, if possible, all other males present in his party, using a Marantz Professional PMD661 solid-state recorder and a Sennheiser ME67 directional microphone. In addition, the context of pant hoot production (travelling or feeding) was noted.

### Data collected and definitions

*Context*. Pant hoots are usually produced in travelling and feeding contexts [42]. Pant hoots given when arriving at a feeding site (e.g. approaching or climbing a feeding tree), or during feeding, were classified as ‘feeding’ pant hoots. We classified pant hoots produced when moving on the ground (as opposed to arriving at a feeding site or feeding) as ‘travel’ pant hoots [42].

*Dominance rank.* This was calculated using the Elo-rating procedure, which is based on sequences of agonistic interactions between individuals [48]; see Additional file 3).

### Selection of recordings and acoustic features

An utterance was defined as a “pant hoot” only if it contained the climax phase [26, 42]. We only considered recordings for analyses if they were of high quality without background noise. As well as the number of calls in each phase and the whole sequence, and the duration of calls, we assessed the F0 of calls (peak frequency in Hz of the F0 at the middle of a call) and phase duration (time in seconds between the start of the first call and the end of the last call of a phase).

### Statistical analyses

We used linear mixed-effect models (LMM) with maximum likelihood estimates using R, version 3.1.2 [49] and the lme 4 package, version 1.0–7 [50]. In models testing for a negative relationship between call duration and number, call duration was the dependent variable, and the number of calls (per phase or in the entire pant hoot utterance) was the test fixed variable. Since behavioural state might affect the acoustic structure of pant-hooting [51], the context of call production (travelling vs. feeding) was included as a control fixed variable. In models testing for a negative relationship between the durations of adjacent phases, the dependent variable was the duration of build-up, climax, or let-down, respectively, and the fixed variable was the duration of the preceding phase (i.e. introduction, build-up, or climax, respectively). The context of call production was entered as a fixed control variable. In this particular analysis we excluded all pant hoots with missing build-up (*N* = 47) or let-down (*N* = 55) phases. In models testing whether, within a phase, call F0 was related to call number or duration, call F0 was the dependent variable, and both call duration and the number of calls in a phase were fixed test variables. In addition to context of call production, age and dominance rank of the caller were entered as control fixed variables, since these two attributes correlate with F0 of pant-hooting. In all our models we entered as random intercept caller ID, together with random slopes for all the fixed variables within individuals. We entered pant hoot ID as another random intercept since we measured multiple calls from the same pant hoot. Recordings with incomplete introduction phases (*N* = 50) were not incorporated in the analyses concerning the introduction and the entire pant hoot.

We used a likelihood ratio test (LRT) to test the full model against a null model (comprising the intercept and random effects) and to test the significance of individual independent variables [52, 53]. There was no collinearity between the examined independent variables (variance inflation factors of the independent variables were below the value of 2). Prior to the analyses, if necessary, variables were transformed to achieve more symmetrical distributions (see Additional files 4 and 5 for details on which transformation type was used for each variable), and values of all quantitative variables were scaled to a mean of 0 and standard deviation of 1. We ran bootstraps to estimate 95% confidence intervals around the estimates of each fixed effect.

Since data from each call within a sequence were used in three different models (two on the phase level and one on the entire pant hoot level), we controlled the Type I error rate by the sequential Bonferroni technique [54, 55], using a Bonferroni adjustment (*k*) equal to 3. Since in the analyses with phase duration data from the build-up and the climax were used twice, we applied a Bonferroni adjustment equal to 2.

## Results

Descriptive statistics for duration, number and F0 of calls in each phase and the entire pant hoot, and for the duration of the phases and overall sequence, are shown in Table 1.

**Table 1:** Mean (±SD) values of call duration, number of calls and call F0, per phase and in the whole pant hoot, and the duration of each phase and the entire sequence

	Introduction	Build-up	Climax	Let-down	Entire pant hoot
Call duration (s)	0.48±0.31	0.21±0.07	0.57±0.24	0.20±0.04	0.37±0.27
Phase duration (s)	5.07±2.10	2.47±1.12	1.20±0.60	1.11±0.82	8.05±3.05
N calls	6.68±3.01	5.78±2.48	2.25±1.00	4.27±2.44	14.61±4.04
Call F0 (Hz)	400.04±180.30	302.17±92.81	1182.67±265.24	339.43±82.28	473.74±340.6

### Is there a negative relationship between call duration and number?

**Fig. 2:**
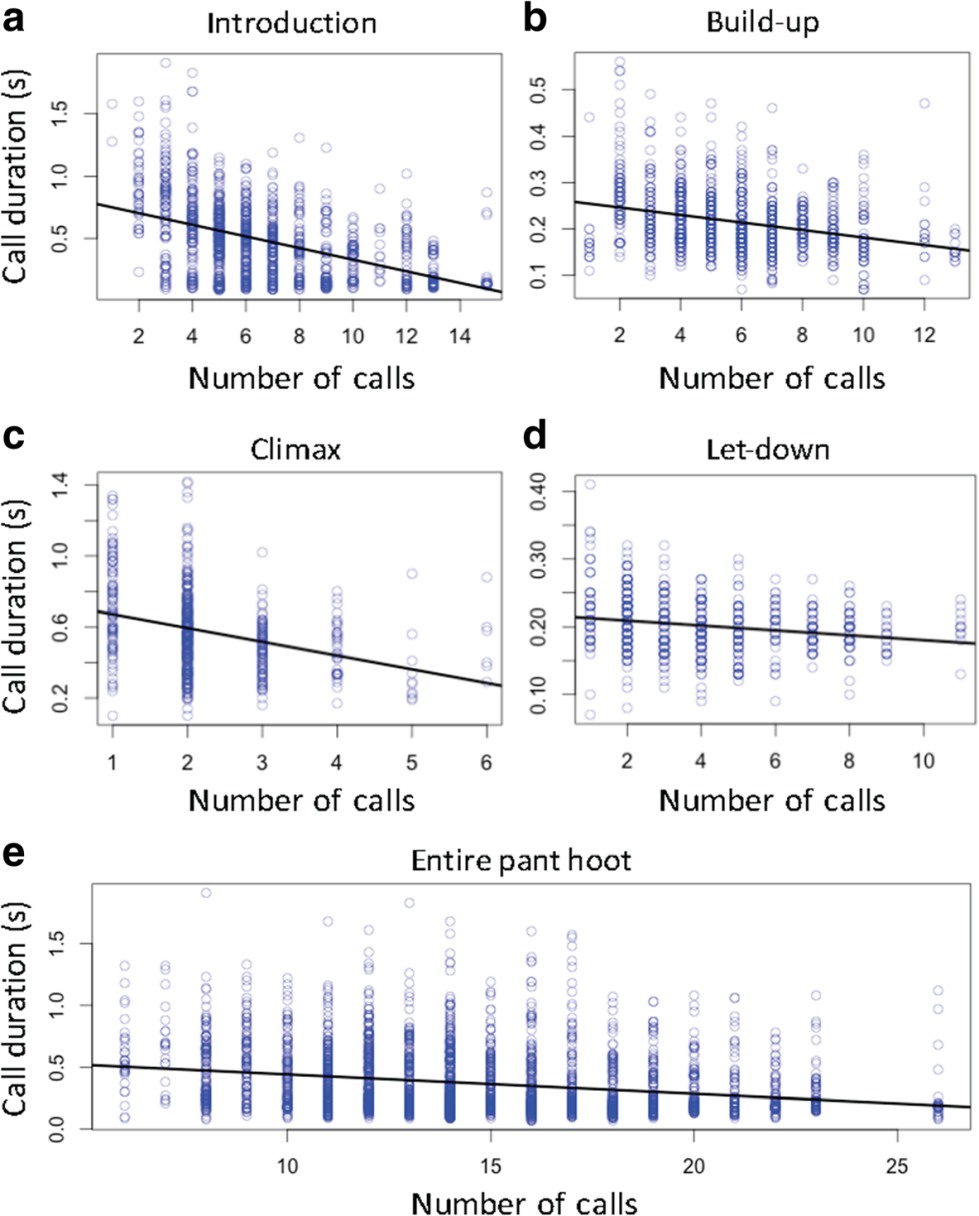
The relationship between call duration and the number of calls in the four phases of a pant hoot and the entire sequence. **a** – introduction, **b** – build-up, **c** – climax, **d** – let-down, **e** – entire pant hoot. Black line represents regression line; circles represent data points

There were significant negative relationships between call duration and the number of calls in all four phases - introduction (Fig. 2a), build up (Fig. 2b), climax (Fig. 2c), let-down (Fig. 2d) - and for the entire pant hoot (Fig. 2e) (Table 2).

**Table 2:** The relationship between call duration and the investigated (fixed) variables in the introduction, build-up, climax, let-down, and entire pant hoot

Introduction				
* Independent variable*	*Estimate ± SE*	*χ* ^*2*^	*p value*	*95% confidence interval*
** Number of calls**	**−0.45**±**0.04**	**19.56**	**<0.001**	**−0.52 to − 0.35**
Context	0.25±0.08	7.03	0.008	0.08 to 0.41
Build-up				
* Independent variable*	*Estimate ± SE*	*χ* ^*2*^	*p value*	*95% confidence interval*
** Number of calls**	**−0.15**±**0.06**	**4.41**	**0.036**	**−0.32 to − 0.01**
Context	−0.03±0.18	0.03	0.857	−0.47 to 0.37
Climax				
* Independent variable*	*Estimate ± SE*	*χ* ^*2*^	*p value*	*95% confidence interval*
** Number of calls**	**−0.32**±**0.07**	**12.54**	**<0.001**	**−0.49 to − 0.14**
Context	0.35±0.11	5.99	0.014	0.09 to 0.57
Let-down				
* Independent variable*	*Estimate ± SE*	*χ* ^*2*^	*p value*	*95% confidence interval*
** Number of calls**	**−0.14**±**0.06**	**10.94**	**<0.001**	**−0.37 to − 0.11**
Context	0.17±0.15	1.11	0.291	−0.14 to 0.55
Entire pant hoot				
* Independent variable*	*Estimate ± SE*	*χ* ^*2*^	*p value*	*95% confidence interval*
** Number of calls**	**−0.25**±**0.03**	**23.16**	**<0.001**	**−0.31 to − 0.19**
Context	0.09±0.06	1.55	0.213	−0.06 to 0.20

Test variables are in bold. (LMM; dependent variable: call duration; random intercepts: pant hoot ID and caller ID)

### Is there a negative relationship between durations of adjacent phases?

**Fig. 3:**
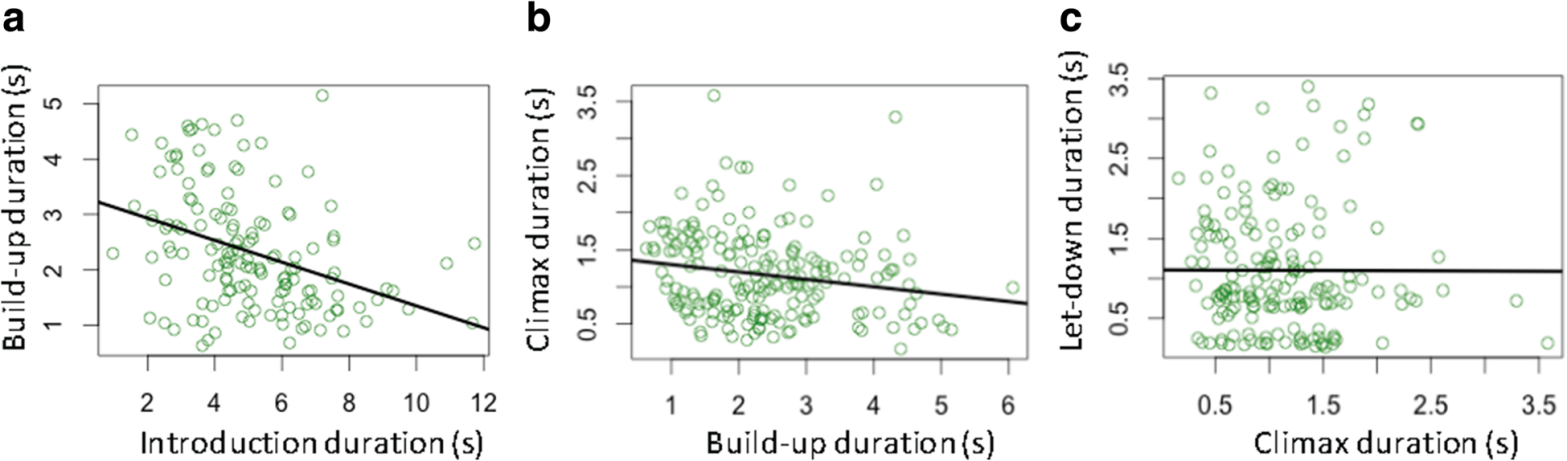
The relationship between the durations of adjacent phases. **a** – introduction and build up, **b** – build-up and climax, **c** – climax and let-down. Black line represents regression line; circles represent data points

There was a significant negative relationship between the duration of the introduction and build-up (estimate ± SE = −0.11 ± 0.04, *χ* ^*2*^ = 5.53, *p* = 0.019, 95% CI = −0.21 to −0.02; Fig. 3a), and of the build-up and climax phases (estimate ± SE = −0.09±0.04, *χ* ^*2*^ = 5.93, *p* = 0.015, 95% CI = −0.18 to −0.02; Fig. 3b). The durations of the climax and the let-down phases were not related (estimate ± SE = −0.08±0.11, *χ* ^*2*^ = 0.52, *p* = 0.469, 95% CI = −0.32 to 0.14; Fig. 3c).

### Is there a relationship between call F0 and call duration?

**Fig. 4:**
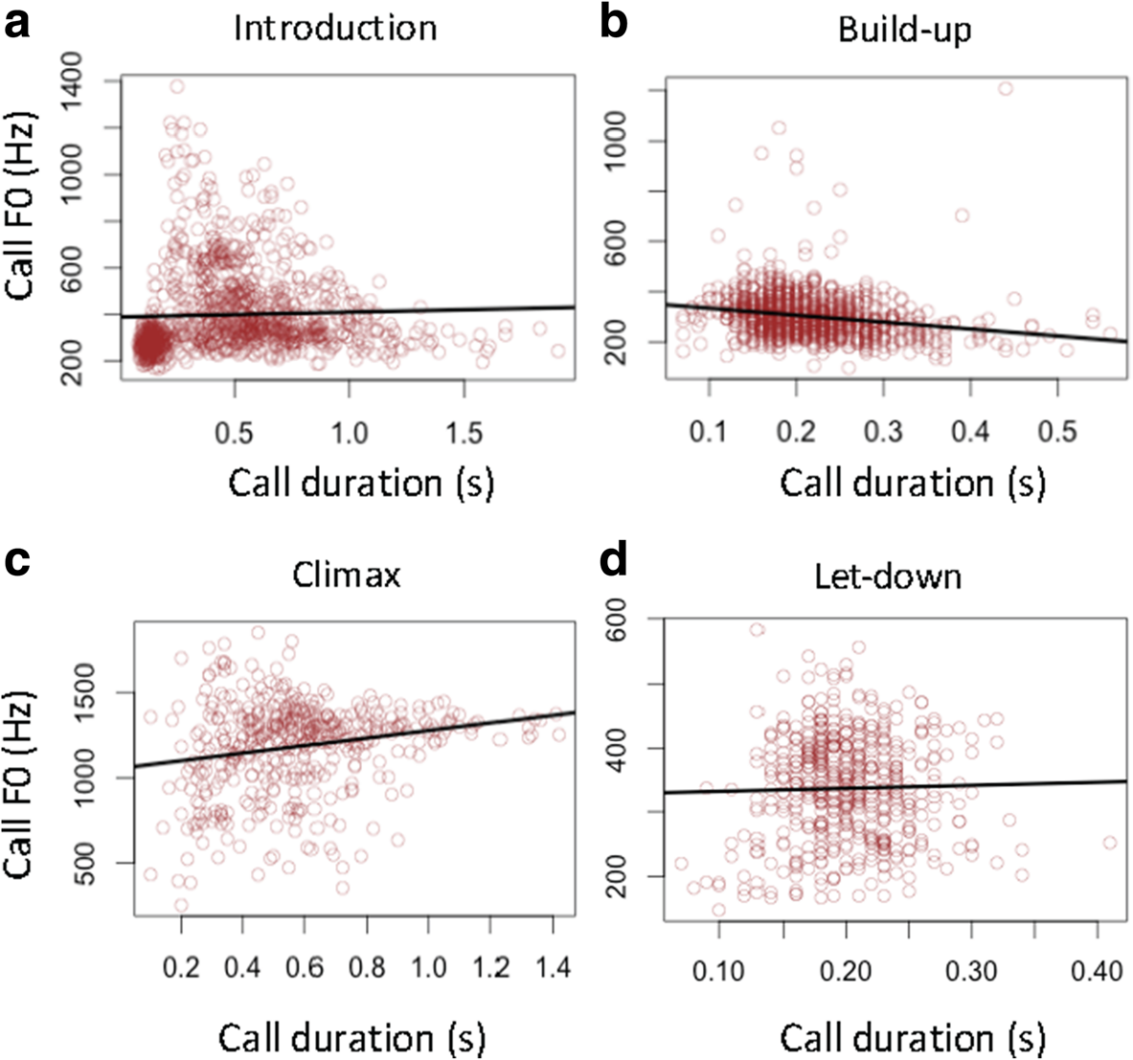
The relationship between call F0 and duration in the four phases of a pant hoot. **a** – introduction, **b** – build-up, **c** – climax, **d** – let-down. Black line represents regression line; circles represent data points

There was a significant positive relationship between call F0 and duration in the climax (Table 3; Fig. 4c) and a significant negative relationship between these two variables in the build-up (Table 3; Fig. 4b). There was no relationship between call F0 and duration in the introduction or let-down phases (Table 3; Fig. 4a and d).

**Table 3:** The relationship between call F0 and the investigated (fixed) variables in the introduction, build-up, climax, and let-down

Introduction				
* Independent variable*	*Estimate ± SE*	*χ* ^*2*^	*p value*	*95% confidence interval*
** Number of calls**	**0.07**±**0.06**	**1.16**	**0.282**	**−0.06 to 0.23**
** Call duration**	**0.06**±**0.12**	**0.28**	**0.595**	**−0.19 to 0.32**
Context	0.23±0.10	3.62	0.057	−0.01 to 0.47
Age	0.18±0.37	0.21	0.647	−0.65 to 1.18
Dominance rank	0.15±0.18	0.64	0.424	−0.27 to 0.58
Build-up				
* Independent variable*	*Estimate ± SE*	*χ* ^*2*^	*p value*	*95% confidence interval*
** Number of calls**	**−0.06**±**0.04**	**2.60**	**0.106**	**−0.16 to 0.02**
** Call duration**	**−0.23**±**0.07**	**6.32**	**0.012**	**−0.38 to − 0.06**
Context	−0.17±0.11	1.72	0.190	−0.45 to 0.12
Age	−0.04±0.10	0.18	0.672	−0.24 to 0.24
Dominance rank	0.06±0.10	0.39	0.533	−0.23 to 0.27
Climax				
* Independent variable*	*Estimate ± SE*	*χ* ^*2*^	*p value*	*95% confidence interval*
** Number of calls**	**0.14**±**0.05**	**6.01**	**0.014**	**0.03 to 0.24**
** Call duration**	**0.31**±**0.07**	**8.61**	**<0.001**	**0.15 to 0.46**
Context	−0.38±0.13	7.53	0.006	−0.73 to −0.12
Age	0.04±0.06	0.43	0.513	−0.09 to 0.31
Dominance rank	0.04±0.13	0.09	0.758	−0.26 to 0.32
Let-down				
* Independent variable*	*Estimate ± SE*	*χ* ^*2*^	*p value*	*95% confidence interval*
** Number of calls**	**0.18**±**0.05**	**9.31**	**0.002**	**0.07 to 0.28**
** Call duration**	**0.04**±**0.07**	**0.27**	**0.600**	**−0.12 to 0.19**
Context	−0.28±0.12	5.45	0.019	−0.57 to −0.05
Age	−0.03±0.12	0.08	0.772	−0.35 to 0.23
Dominance rank	0.02±0.06	0.12	0.726	−0.17 to 0.17

Test variables are in bold. (LMM; dependent variable: fundamental frequency; random intercepts: pant hoot ID and caller ID)

### Is there a relationship between call F0 and call number?

**Fig. 5:**
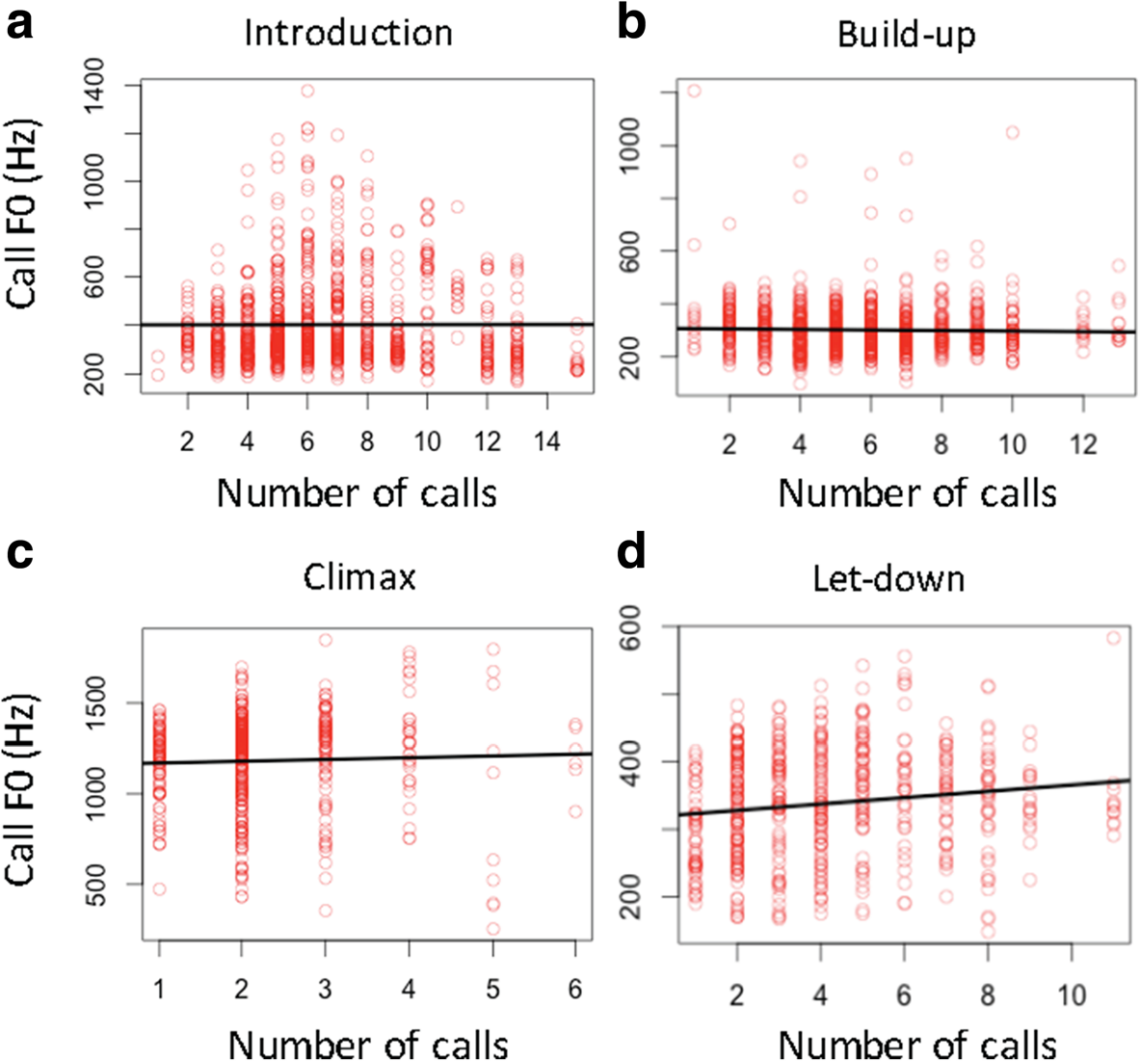
The relationship between call F0 and number in the four phases of a pant hoot. **a** – introduction, **b** – build-up, **c** – climax, **d** – let-down. Black line represents regression line; circles represent data points

There was a positive relationship between call F0 and the number of calls in the climax and let-down (Table 3; Fig. 5c and d). There was no relationship between these variables in the introduction or build-up phases (Table 3; Fig. 5a and b).

## Discussion

In this study of wild chimpanzee pant hoots, we found negative relationships between the number and duration of calls, both at the level of phases within the pant hoot, and for the entire vocal sequence. Negative relationships were also found between the durations of some adjacent phases, namely introduction and build-up, and build-up and climax. While relationships were found in some phases between call F0 and either the number of calls or their durations, the direction of these associations varied between phases. These results imply that there are trade-offs in terms of duration at two levels in pant hoot production - between call number and duration, and between relative duration of successive phases - and that trade-offs between fundamental frequency and call number or duration also occur, with the nature of these being phase-specific.

Our finding of strong, negative relationships between the number of calls and their durations provides further evidence that Menzerath’s linguistic law, which reflects the principle of compression, holds in the vocal communication of non-human animals, adding to similar recent evidence from a study of male gelada call sequences [20]. Importantly, agreement with Menzerath’s law here was seen both in phases with relatively long constituent calls (introduction and climax), and in those with shorter constituent calls (build-up and let-down), implying that compression acts similarly across the distinct parts of pant hoots, regardless of the relative length of constituent calls.

Previous studies have proposed that patterns consistent with compression may be less likely to emerge in situations where vocal signals are directed at distant audiences [20, 24]. For example, in female Barbary macaques, copulation call sequences given around the time of ovulation contain more calls than sequences given early in the cycle, but these calls are longer - not shorter - in duration than those in early cycle sequences [56]. It has been proposed that this pattern may be due to the fact that in this type of long-range communication (female copulation calls appear to function to attract males from large distances), there is a conflict between compression and transmission success, with pressure for the latter being more important [20, 24]. Our results, however, indicate that compression can play an important role in shaping long-distance vocal communication. In pant hoots, the negative relationship between the number and duration of calls was present both in high-amplitude phases, such as the climax (directed, at least in part, at distant receivers) and in low-amplitude phases, such as the introduction (directed primarily at nearby individuals).

In addition to a negative relationship between call number and duration in pant hoots, we found evidence that the durations of particular phases within this vocal sequence depend on the duration of the adjacent phases. Specifically, there was a negative correlation between the duration of the introduction and the build-up, and between the duration of the build-up and the climax. These results imply trade-offs in investment into different phases. Previous analyses of pant hoots suggest that prolonging the duration of particular phases, such as the build-up or the climax, may be used as effective territorial displays or to coordinate chorusing [29, 43]. However, it appears that, in some cases, if one phase is longer in total duration, the subsequent one tends to be shorter; thus, plasticity in phase duration appears somewhat constrained at a broader level. A lack of significant relationship between the durations of the two last phases in the pant hoot - climax and let-down – may be due to the let-down not having a following phase, such that constraints on its duration are relaxed. Many vocal sequences, across a wide range of taxa, are comprised of specific phases or notes produced in a conservative order [11, 12, 25, 57–59]; these provide the opportunity to test the generality of trade-offs in investment between different parts of the sequence.

Together, the results of analyses of call and phase duration indicate that there are trade-offs at two levels in pant hoot production: between call number and call length (if more calls are given, these tend to be shorter in length; or, if longer calls are given, these tend to be fewer in number), and between relative allocation of acoustic activity into subsequent phases (if one phase is longer, the subsequent one tends to be shorter). Theoretical analyses of communication indicate that reducing signal duration decreases transmission fidelity [60], so it is likely that the patterns seen here in pant hoots reflect a compromise between pressure to maximise efficacy of communication and constraints imposed by the energetic demands of producing extended vocal sequences [13–15], biomechanical constraints relating to lung capacity and airflow control [17, 18], or associated breathing-related limitations [16, 19, 61].

Our examination of potential links between call F0 and call number or duration revealed a number of significant relationships, which varied between phases. A strong positive relationship between call F0 and duration was seen in the climax, and a strong negative relationship was seen in the build-up, while no relationship was seen in the introduction or let-down. These findings suggest that, across these different phases, separate trade-offs are (or are not) occurring between pitch and calling effort. For example, the positive relationship between call duration and F0 in the climax indicates that individual calls can either be short and low-pitched or long and high-pitched. In mammals, F0 is mediated by sub-glottal air pressure generated in the lungs, with higher air pressure generating higher F0 as a result of an increased rate of vocal fold vibrations [37]. Our result, therefore, might be a by-product of differences in sub-glottal air pressure, with higher air pressures generating calls that are both longer and higher-pitched. This would indicate that chimpanzees have limited active control over the movement of their larynx, very much in contrast to humans who are able to produce a stable F0 during speech production, more or less independent of sub-glottal air pressure [37, 62]. The negative relationship between call duration and F0 in the build-up may be due to the fact that calls in this phase are much shorter than in the climax; it is possible that there is a critical threshold of call length, above which pitch inevitably rises due to the link with sub-glottal air pressure, but that this threshold is not reached in the build-up phase.

At a functional level, the different relationships between call F0 and duration found in different phases suggest that specific phases within a pant hoot have distinct functions modulated by their pitch [43]. For example, the low-frequency build-up phase seems to be directed (at least in part) to the nearby individuals, since callers adjust its duration depending on the vocal response of the nearby males [29]. The high-frequency high-amplitude climax, on the other hand, seems to be directed at distant receivers [63] and may be an honest signal of individual quality [44]. According to the “calling at the edge” hypothesis [45], mammals calling at near maximum F0 struggle to maintain a harmonic F0, since calling at such extreme frequencies distorts F0 harmonics, resulting in non-linear phenomena (i.e. non-linearity in the vocal fold dynamics) [64]. Indeed, non-linear phenomena are considerably more common in the loud high-frequency climax phase of the pant hoot than in the quieter low-frequency introduction [64]. Calling at maximal frequencies may signal caller quality, since individuals in better biological condition are more likely to produce climaxes that are free from non-linear phenomena (e.g. [45]).

Analysis of call F0 and call number again revealed differences between phases: in only two phases was a clear link found between these variables– a significant positive relationship in the let-down and the climax. Overall, our results in relation to F0 seem to reflect the literature showing inconsistent relationships between call F0 and temporal features. For example, a positive relationship between F0 and both call duration and sequence length was found in chimpanzee victim screams [65]. Similarly, baboon grunts produced in strongly affective situations are both longer and higher frequency than grunts produced in more relaxed situations [66]. In contrast, calls with lower F0 tend to be also longer in Japanese quails [31], while in domestic dogs (*Canis familaris*) [67] F0 and duration of aggressive barks are not correlated. Data from a range of animals, therefore, indicate that there is no elemental, over-arching trade-off between temporal features of call sequences and F0 of the constituent calls; that diverse, context-specific trade-offs may be important merits future research.

## Conclusions

Identifying the basic patterns of organisation of animal signals can provide important insights into the relationship between their structure and function [2, 68, 69] and can also shed light on the fundamental principles underpinning signal evolution [20, 24]. In this study we focussed on the relationship between temporal and spectral variables of wild chimpanzee pant hoots. Our results suggest that costs and constraints involved in vocal production, balanced against the potential benefits to signallers accrued from variation in signal form, lead to trade-offs of multiple kinds. This study highlights the key role that such costs and constraints can play in shaping the temporal and acoustic structure of animal vocal sequences.

## Additional files

Additional file 1: An audio recording of a pant hoot given by an adult male. (WAV 1077 kb)

Additional file 2: Table with estimated age and the dominance rank of the study males, and the number of recordings per male. (DOCX 52 kb)

Additional file 3: Calculation of the dominance status of the study males. (DOCX 107 kb)

Additional file 4: Table with the type of variable transformation in models concerning the relationship between call duration and the investigated (fixed) variables in the introduction, build-up, climax, let-down, and the entire sequence. (DOCX 47 kb)

Additional file 5: Table with the type of variable transformation in models concerning the relationship between call F0 and the investigated (fixed) variables in the introduction, build-up, climax, and let-down. (DOCX 49 kb)
