# Supplementary material for: Trade-offs in the production of animal vocal sequences: insights from the structure of wild chimpanzee pant hoots
Source: Front Zool. 2017 Nov 6;14:50. doi: 10.1186/s12983-017-0235-8 (PMC5674848; doi:10.1186/s12983-017-0235-8)
Supplement: Supplementary file 2 — Table with estimated age and the dominance rank of the study males, and the number of recordings per male. (DOCX 52 kb) [file 12983_2017_235_MOESM2_ESM.docx]

| Male ID | Age (years) | Rank | Number of pant hoot recordings |
| --- | --- | --- | --- |
| FD | 19 | 7 | 8 |
| FK | 14 | 6 | 25 |
| HW | 20 | 2 | 31 |
| KT | 18 | 5 | 11 |
| KZ | 18 | 12 | 24 |
| MS | 22 | 1 | 23 |
| NK | 31 | 4 | 24 |
| PS | 15 | 10 | 26 |
| SQ | 22 | 3 | 16 |
| SM | 20 | 11 | 1 |
| ZF | 31 | 9 | 18 |
| ZG | 16 | 13 | 2 |
| ZL | 18 | 8 | 33 |
| Total |  |  | 242 |

**Additional File 2:** Estimated age and the dominance rank (1= most dominant) of the study males, and the number of recordings per male
