# Supplementary material for: Trade-offs in the production of animal vocal sequences: insights from the structure of wild chimpanzee pant hoots
Source: Front Zool. 2017 Nov 6;14:50. doi: 10.1186/s12983-017-0235-8 (PMC5674848; doi:10.1186/s12983-017-0235-8)
Supplement: Supplementary file 4 — Table with the type of variable transformation in models concerning the relationship between call duration and the investigated (fixed) variables in the introduction, build-up, climax, let-down, and the entire sequence. (DOCX 47 kb) [file 12983_2017_235_MOESM4_ESM.docx]

**Additional File 4:** Type of variable transformation in models concerning the relationship between call duration and the investigated (independent) variables in the introduction, build-up, climax, let-down, and the entire sequence

| VariabledVaVa Variable | *Introduction* | *Build-up* | *Climax* | *Let-down* | *Entire sequence* |
| --- | --- | --- | --- | --- | --- |
| Call duration | Log | Log | None | None | Log |
| Number of calls | Square-root | Square-root | None | None | Square-root |
| Context | None | None | None | None | None |
