# Supplementary material for: Trade-offs in the production of animal vocal sequences: insights from the structure of wild chimpanzee pant hoots
Source: Front Zool. 2017 Nov 6;14:50. doi: 10.1186/s12983-017-0235-8 (PMC5674848; doi:10.1186/s12983-017-0235-8)
Supplement: Supplementary file 5 — Table with the type of variable transformation in models concerning the relationship between call F0 and the investigated (fixed) variables in the introduction, build-up, climax, and let-down. (DOCX 49 kb) [file 12983_2017_235_MOESM5_ESM.docx]

Additional File 5: Type of variable transformation in models concerning the relationship between call F0 and the investigated (independent) variables in the introduction, build-up, climax, and let-down

| Variable | *Introduction* | *Build-up* | *Climax* | *Let-down* |
| --- | --- | --- | --- | --- |
| Call F0 |  | Log | None | None |
| Number of calls | None | Square-root | Log | Log |
| Call duration | None | Log | Square-root | Log |
| Context | None | None | None | None |
| Age | None | Log | Square-root | Square-root |
| Dominance rank | None | None | None | None |
